# Genetic Correlations among Canine Hip Dysplasia Radiographic Traits in a Cohort of Australian German Shepherd Dogs, and Implications for the Design of a More Effective Genetic Control Program

**DOI:** 10.1371/journal.pone.0078929

**Published:** 2013-11-07

**Authors:** Bethany J. Wilson, Frank W. Nicholas, John W. James, Claire M. Wade, Herman W. Raadsma, Peter C. Thomson

**Affiliations:** Faculty of Veterinary Science, The University of Sydney, Sydney, New South Wales, Australia; University of Queensland, Australia

## Abstract

Canine hip dysplasia (CHD) is a common musculoskeletal disease in pedigree dog populations. It can cause severe pain and dysfunction which may require extensive medication and/or surgical treatment and often ultimately requires humane euthanasia. CHD has been found to be moderately heritable and, given its impact on welfare, should be considered an imperative breeding priority. The British Veterinary Association/Kennel Club scoring method is one of several measures used to assess the genetic propensity of potential breeding stock for dysplastic changes to the hips based on radiographic examination. It is a complex measure composed of nine ordinal traits, intended to evaluate both early and late dysplastic changes. It would be highly desirable if estimated breeding values (EBVs) for these nine traits were consolidated into a simpler, EBV-based, selection index more easily usable by breeders. A multivariate analysis on the phenotype scores from an Australian cohort of 13,124 German Shepherd Dogs (GSDs) returned genetic correlations between 0.48–0.97 for the nine traits which fell into two trait groups, Group 1 reflecting early changes (“laxity”) and Group 2 reflecting late changes (“osteoarthritis”). Principal components analysis of the ordinal EBVs suggested the same pattern, with strong differentiation between “laxity” and “osteoarthritis” traits in the second component. Taking account of all results, we recommend interim use of two selection indexes: the first being the average of ordinal EBVs for “laxity” traits and the second being the average of ordinal EBVs for “osteoarthritis” traits. The correlation between these two selection indexes (0.771–0.774) is sufficiently less than unity enabling the selection of dogs with different genetic propensity for laxity and for osteoarthritic CHD changes in GSDs; this may also be applicable in other breeds. Dogs with low propensity for severe osteoarthritic change in the presence of laxity may be of interest both in molecular research and breeding programs.

## Introduction

Canine hip dysplasia (CHD) is a common inherited musculoskeletal disease of dogs. CHD is characterized by an excessive degree of laxity of the coxofemoral joint at birth which causes the hips, otherwise anatomically normal, to accumulate varying degrees of damage due to abnormally distributed joint forces, resulting in a dysplastic and arthritic hip. The disease is usually bilateral, although some difference in severity may be noted between the right and the left hip [1]–[3]. The mode of inheritance is considered to be multifactorial with both a suite of genes and environmental factors contributing to the occurrence and severity of the disease [1], [4], [5]. Several studies have been suggestive of a major gene [6]–[9] and various QTLs and SNPs have been identified as potentially associated with the disorder [10]–[16].

Various control schemes have been established to screen potential breeding candidates with the aim of orchestrating some genetic control over the disease [17] which would complement modification of environmental factors such as diet and weight management. Such schemes typically involve radiographic examination of the hip joint followed by an attempt to quantify the degree of disease which is exhibited at the time of observation. The method of CHD scoring that has been most extensively used in Australia is based on the British Veterinary Association (BVA)/Kennel Club (KC) scheme and, following the UK in Australia, by the AVA/ANKC (Australian Veterinary Association/Australian National Kennel Club) scheme. The BVA/KC Scheme evaluates nine traits for each hip (referred to in this paper as British Veterinary Association Hip Traits – BVAHTs) assessed against an ordinal categorical scale from a radiograph taken with the hips in extension. Assessing each hip individually, Norberg Angle (NORB), Subluxation (SUBL), Cranial Acetabular Edge (CrAE), Dorsal Acetabular Edge (DAE), Cranial Effective Acetabular Rim (CrEAR), Acetabular Fossa (AF), Femoral Head and Neck Exostosis (FHNE), and Femoral Head Remodelling (FHR) are each placed into one of seven categories labelled with a number between 0 (normal) and 6 (greatest deviation from normal). One trait (Caudal Acetabular Edge – CaAE) is placed into one of six categories labelled from 0 to 5 [17]–[19]. The exact way in which Australian breeders have attempted in practice to utilize the complicated construction of BVAHT scores to breed against hip dysplasia is not well understood. The pathological changes associated with each BVAHT score are described in detail elsewhere [18].

Despite the use of this scheme in Australia, and several other countries with substantial pedigree dog populations, the BVA/KC scheme phenotype has not been utilized optimally to control hip dysplasia as is argued below and by others [20], [21]. A previous study of a cohort of Australian-born German Shepherd Dogs demonstrated very high genetic correlations between right and left BVAHTs (on the underlying liability scales), indicating left and right BVAHTs are effectively the same traits, genetically [3]. Subsequent papers on the same data set reported the heritability of the nine traits within this cohort,while accounting for the ordinal nature of the traits [5], and demonstrated the feasibility of estimated breeding values (EBVs) derived using several methods of analysis of the trait phenotypes, allowing more accurate selection for these traits [22]. In the latter paper the authors concluded that EBVs for BVAHTs calculated via ordinal logistic regression were the optimal choice for EBV-based selection in the studied cohort of Australian German Shepherd Dogs. EBVs calculated by fitting a linear model to individual BVAHTs or a single EBV calculated by fitting a linear model to the summed BVAHT scores were found to be suboptimal due to unwarranted assumptions about the relationship of BVAHT scores to the true underlying variation. While correlations between linear and ordinally-derived EBVs for BVAHT scores were high, the relationship between the fitted regression values and the standardized residuals appeared to be non-random (and non-linear), suggesting that linear-derived EBVs are not a good proxy for the more methodologically correct ordinally-derived EBVs. As EBVs derived from the summed scores of the BVAHT involve all the same assumptions as the linear analysis of single BVAHTs (and additional assumptions about the relationship between BVAHTs), this type of EBV was also deemed less suitable.

While the previous finding [5] allows us to confidently combine left and right scores into a single EBV, derived from an ordinal analysis for each BVAHT, this would still leave breeders with nine different EBVs to consider simultaneously. For ease of use by end-users, i.e. breeders and breed societies, it would be advantageous if the ordinal EBVs could be effectively combined into a single selection index ([23];p327–328). Ideally, such a process would be undertaken with an understanding of how the genetic component of each BVAHT correlates to the genetic component of the goal of selection (a lifetime of favourable welfare with respect to CHD). However, in the absence of this information it is still possible to examine the correlation structure of the breeding-value component of the nine BVAHTs, and use this knowledge in the design of a more effective selection program against CHD to be used while further information is gathered and used to form a basis into which new information can be easily incorporated as it is validated.

The purpose of this paper is, therefore, to explore the correlations among the ordinal EBVs for BVAHTs, and to determine a simple but methodologically defensible interim selection index of no more than two EBVs for selection against CHD in this population. A previous paper described how NORB, SUBL and CrAE (“laxity” traits) are substantially more phenotypically variable than the remaining traits (“osteoarthritic” traits) in a large cohort of Australian German Shepherd dogs born between 1976 and 2005 [5]. This result was similar to the phenotypic variation observed in a large study of Labradors in the United Kingdom [21]. Given the accepted pathogenesis of CHD as osteoarthritic change caused by excessive joint laxity, this pattern of variation could be suggestive of two underlying traits, representing a heritable degree of joint laxity and also a heritable degree of osteoarthritic change in response to a given degree of joint laxity. We will explore this relationship by estimating genetic correlations between the BVAHTs and examining the relationship between ordinally-derived EBVs for BVAHTs using principal component analysis.

## Materials and Methods

### Data

Two sources of BVAHT data were used in this study, namely (1) data accumulated by Dr Malcolm Willis in the United Kingdom from records collected within the Australian Veterinary Association/Australian National Kennel Council (AVA/ANKC) canine hip and elbow dysplasia scheme (CHEDS) and the records of radiologists sent to him privately; and (2) data supplied by the German Shepherd Dog Council of Australia (GSDCA) hip dysplasia breed scheme. Pedigree information regarding Australian GSDs held by the ANKC was supplied with permission of the GSDCA by Dogs NSW. All data sets included all data available electronically at the time at which the records were obtained. The data set contains the animal's name, pedigree information, year of birth, age at radiographic study, sex and scores for each of the 18 BVAHTs.

The final combined data set comprises of a pedigree record and a single complete BVAHT record set from 13,124 (8,793 female, 4,331 male) German Shepherd Dogs born between 1976 and 2006. The median age at the time of radiographic study was 19 months. A full account of the assembly of this data set and details of the score distributions has been published previously [22].

In addition to the raw scores, EBVs for these dogs, calculated using a multi-threshold model in ASReml 3 using methodology previously reported [22], were used as input data for a principal component analysis.

### Genetic correlation

Estimation of the genetic correlation between traits requires estimation of the genetic variances of these traits as well as the calculation of the genetic covariance between them. Simultaneous multivariate analysis of nine traits is computationally difficult, leading the authors to instead construct the genetic variance-covariance matrix using the results of bivariate analyses of each pair combination of the nine BVA hip traits. As there was no method within ASReml at the time of the study for multivariate analysis of ordinal data, a linear mixed model was used of the form
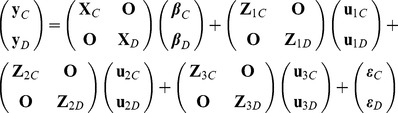
 where vectors **y** *_C_* and **y** *_D_* represent the hip scores (phenotypes) from BVA hip traits *C* and *D*, respectively, of 2*n* hips (two hips for each of *n* dogs). **β** *_C_* and **β** *_D_* are vectors of the fixed effects which are related to **y** *_C_* and **y** *_D_* by the model matrices **X** *_C_* and **X** *_D_*. Similarly, ***u*** *_iC_* and ***u*** *_i_*_D_ (*i* = 1, 2, 3, described below) are vectors of random effects which are related to **y** *_C_* and **y** *_D_* by the incidence matrices **Z** *_iC_* and **Z** *_iD_* (i = 1, 2, 3), while **ε** *_C_* and **ε** *_D_* are vectors of random residual effects.

The fixed effects specified for the two traits in all the bivariate models are the hip side (left or right), the sex of dog, the year in which the dog was born, and the age of the dog at the time that the radiograph was taken (covariate). The random effects in each model are the additive genetic effect of the dog (*i* = 1), the permanent environmental effect of the dog (*i* = 2) which links left and right side scores together, and a litter environmental effect (*i* = 3). Note that there will be separate effects for each trait considered, in each bivariate analysis. Also, because the same explanatory model terms were used for both traits in any model, as well as the same animal configurations, **X** *_C_* = **X** *_D_* and **Z** *_iC_* = **Z** *_iD_* (*i* = 1, 2, 3) in these analyses.

In order to estimate the genetic correlation between **y** *_C_* and **y** *_D_*, we wish to obtain an estimate of the 2×2 genetic variance-covariance matrix (VCVM) 
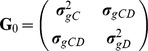
 where 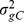
and 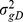 are the additive genetic variances of BVAHTs *C* and *D* respectively, and 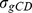 is the additive genetic covariance between BVAHTs *C* and *D*. To obtain environmental and phenotypic correlations, we also need to estimate the corresponding 2×2 VCVM of the residual random error terms, **R**_0_, with similar corresponding elements. ASReml [24] estimates these variance and covariance parameters using a Restricted Maximum Likelihood (REML) method developed by Patterson and Thompson [25].

### Matrix bending

Covariances were all estimated by the corresponding bivariate analysis and variances by a linear univariate analysis using the same fixed and random effects. Due to the piecemeal construction of the VCVMs from separate bivariate and univariate analyses, there was a possibility that the matrix would have negative eigenvalues, meaning the matrix was neither positive definite nor positive semi-definite and therefore invalid. To address this problem, we calculated the eigenvalues of the matrices, and in the case of one or more eigenvalue being negative, we performed a bending procedure as described in Gunawan and James [26], based on the method proposed by Hayes and Hill [27].

Let ν_1_, ν_2_, …, ν*_t_* be the eigenvalues of the matrix **P** ^−1^ **G** sorted from smallest to largest, where **P** represents the phenotypic VCVM and **G** represents the genetic VCVM and *t* represents the number of traits in the matrix, in this case the nine BVAHTs. If 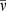
represents the mean eigenvalue, then the bending factor (1 – *λ*) to be applied to **G** is
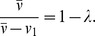



The **G** matrix corrected by bending (G*) was obtained by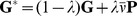 as a valid **P** could be estimated directly from the score data.

Once estimates for these parameters were obtained, the genetic correlation (*r*_g_) was estimated as:
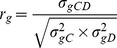



### Principal Component Analysis of EBVs (vs averages of EBVs)

Principal component analyses (PCA) of both the raw data and the EBVs from ordinal analyses [22] were calculated to compare any patterns of association between BVAHTs which appeared in the genetic and phenotypic VCVMs. The principal component analysis was undertaken in R statistical software version 2.8. It was assumed that all the BVAHTs were being measured using the same units and that difference in the variance between traits was genuine. Therefore, PCA was performed using the VCVM rather than the correlation matrix.

## Results

### Pre-bending matrix (G)

The genetic VCVM constructed directly from the variance and covariance components estimated from the bivariate and univariate analyses had three negative eigenvalues out of nine prior to bending. As a result, bending was undertaken, with a calculated bending factor of approximately 0.11 to create a positive definite matrix. ν_9_, the largest eigenvalue of **P** ^−1^ **G**, was not greater than one prior to bending, so it was not necessary to adjust the calculated bending factor to shrink eigenvalues of **P** ^−1^ **G** below unity.

### Post-bending matrix (G*)

**Figure 1:**
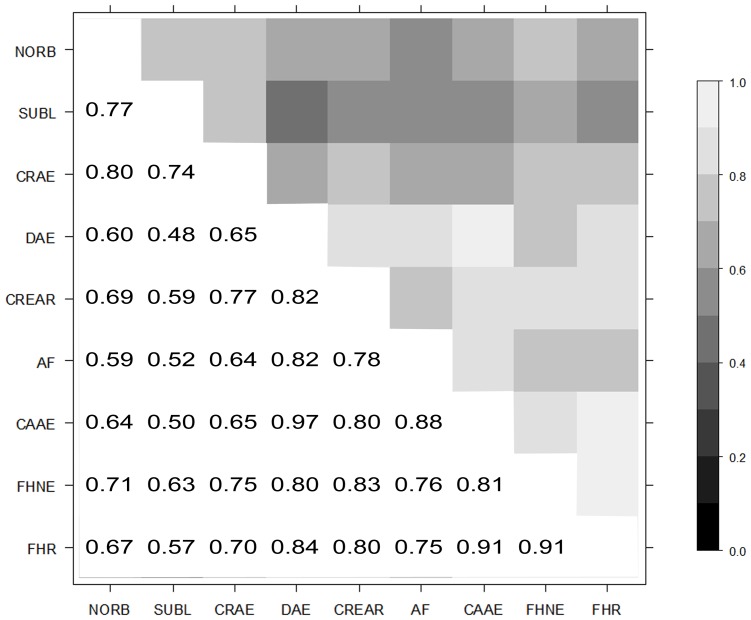
Genetic correlation matrix of British Veterinary Association hip traits (BVAHTs). NORB =  Norberg Angle, SUBL =  Subluxation, CrAE =  Cranial Acetabular Edge, DAE =  Dorsal Acetabular Edge, CrEAR =  Cranial Effective Acetabular Edge, AF =  Acetabular Fossa, CaAE =  Caudal Acetabular Edge, FHNE =  Femoral Head and Neck Exostosis, and FHR =  Femoral Head Remodelling.

The bending procedure resulted in a valid G* with nine positive eigenvalues and a corresponding correlation matrix with all values within the range of −1 to 1. The corrected correlation matrix is shown in Figure 1.

As demonstrated in Figure 1, the genetic correlations between the BVAHTs are moderately high and positive, ranging between 0.48 and 0.97. “Laxity” traits (NORB, SUBL and CrAE) had an average within-group genetic correlation of 0.77 and an average genetic correlation of 0.63 with the remaining (“osteoarthritis”) traits. “Osteoarthritis” traits had a within-group average genetic correlation of 0.83. This finding is similar to the correlations of EBVs from separate ordinal analyses for these traits previously reported [22]. The larger correlations within groups than between them suggests the possibility that BVAHTs may capture information from (at least) two major underlying genetic processes, one relating to early morphological changes and/or laxity and one relating to secondary changes or osteoarthritis (or to look at it another way, the same multifactorial, pathological changes are being characterized by more than one BVAHT).

### Principal component analysis of estimated breeding values

The loadings from the PCA of the ordinal-analysis based EBVs (Table 1) demonstrate a similar pattern to that observed in the genetic correlations. After the “averaging effect” of the first component (73.7% of the variance), the second most important source of variation (component 2), accounting for approximately 10% of the variance, was the difference between “laxity” and “osteoarthritis” traits, reflecting the higher intra-group genetic correlations (0.77 and 0.83) compared to the intergroup genetic correlations (0.63) reported above. Following this finding, the data set was divided into “laxity” and “osteoarthritis” EBVs and separate PCAs were performed to determine the best linear weightings (component one) for each group (see Table 2). This first component accounted for 84.7% and 80.9% of the variance for “laxity” and “osteoarthritis” traits, respectively. Each of the remaining components (which by definition are uncorrelated to and independent of preceding components) accounted for less than 10% of the variance in both analyses. The most important of the remaining components in the “laxity” group analysis delineated NORB and SUBL from CrAE (8.6% of variance). In the “osteoarthritis” group analysis, the next most important component grouped DAE, AF and CaAE separately from CrEAR, FHNE and FHR (7.5% of variance).

**Table 1:** Principal component analysis loadings from an analysis of EBVs for nine British Veterinary Association hip traits (BVAHTs) calculated using ordinal logistic regression.

	Comp.1	Comp.2	Comp.3	Comp.4	Comp.5	Comp.6	Comp.7	Comp.8	Comp.9
**NORB**	−0.400	−0.555	−0.340	0.612	0.147	−0.084	0.111	0.012	0.012
**SUBL**	−0.450	−0.305	−0.372	−0.677	−0.279	0.121	−0.075	−0.052	−0.086
**CrAE**	−0.429	−0.243	0.747	−0.126	0.365	0.157	-0.152	−0.008	0.041
**DAE**	−0.198	0.273	−0.049	0.210	−0.135	0.393	-0.116	−0.784	0.197
**CrEAR**	−0.305	0.274	0.127	0.051	−0.169	0.262	0.803	0.168	−0.207
**AF**	−0.284	0.456	−0.347	−0.173	0.714	−0.201	0.042	−0.001	0.092
**CaAE**	−0.249	0.319	−0.064	0.248	−0.103	0.224	−0.520	0.304	−0.589
**FHNE**	−0.326	0.179	0.213	0.049	−0.339	−0.798	−0.015	−0.223	−0.113
**FHR**	−0.268	0.217	−0.006	0.103	−0.292	0.057	−0.169	0.461	0.735
**Standard deviation**	1.61	0.60	0.41	0.34	0.32	0.28	0.22	0.18	0.16
**Variation explained**	73.6%	10.2%	4.8%	3.3%	2.9%	2.2%	1.4%	0.9%	0.7%

The signs associated with the loadings are arbitrary in the sense that the signs of all the loadings within any column can be switched. NORB =  Norberg Angle; SUBL =  Subluxation; CrAE =  Cranial Acetabular Edge; DAE =  Dorsal Acetabular Edge; CrEAR =  Cranial Effective Acetabular Rim; AF =  Acetabular Fossa; CaAE =  Caudal Acetabular Edge; FHNE =  Femoral Head and Neck Exostosis; FHR =  Femoral Head Remodelling.

**Table 2:** Principal component analysis loadings from Components 1 and 2 of separate analysis of EBVs for Group 1 and Group 2 British Veterinary Association hip traits (BVAHTs) calculated using ordinal logistic regression.

	Component 1	Component 2
**Group 1 Principal Component Analysis**		
**NORB**	−0.564	0.515
**SUBL**	−0.600	0.281
**CrAE**	−0.567	−0.810
**Group 2 Principal Component Analysis**		
**DAE**	−0.306	0.026
**CrEAR**	−0.446	−0.169
**AF**	−0.445	0.819
**CaAE**	−0.381	0.039
**FHNE**	−0.461	−0.508
**FHR**	−0.390	−0.201

The signs associated with the loadings are arbitrary in the sense that the signs of all the loadings within any column can be switched. NORB =  Norberg Angle; SUBL =  Subluxation; CrAE =  Cranial Acetabular Edge; DAE =  Dorsal Acetabular Edge; CrEAR =  Cranial Effective Acetabular Rim; AF =  Acetabular Fossa; CaAE =  Caudal Acetabular Edge; FHNE =  Femoral Head and Neck Exostosis; FHR =  Femoral Head Remodelling.

### Correlation between PCA estimates and average estimates of EBVs

**Figure 2:**
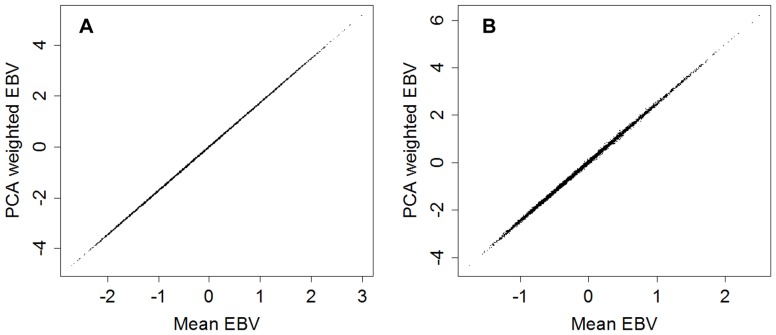
Correlations between the arithmetic mean estimates and first component principal component analysis estimates of the laxity (A) and osteoarthritis (B) estimated breeding values.

Identification of stronger genetic correlations within the “laxity” group and the “osteoarthritis” group traits, reflected in the PCA of EBVs, suggested that two sets of EBVs may represent an acceptable compromise between complexity and comprehensiveness in construction of a selection index based upon BVAHTs. Therefore, “laxity” and “osteoarthritis” EBVs were assembled both by a simple arithmetic mean of a dog's EBVs for the traits from each group and also by use of the best linear weightings calculated from the first component of the PCA from each group. The “laxity” group represents changes which tend to appear earlier in the disease process and the “osteoarthritis” group represents changes which tend to appear later in the disease process. For simplicity and to reflect the underlying aetiology of laxity leading to degenerative change, EBVs for these traits are referred to as “Laxity” and “Osteoarthritis” EBVs, although it is worth noting that degenerative change forms a portion of phenotypic variation estimated by the “laxity” group. EBVs calculated by each of these methods represent an estimate of the true breeding values for these underlying traits. An indication of the correlation of EBVs estimated by these methods is presented in Figure 2. Both correlation coefficients are in excess of 0.999.

### Genetic trends of combined EBVs

**Figure 3:**
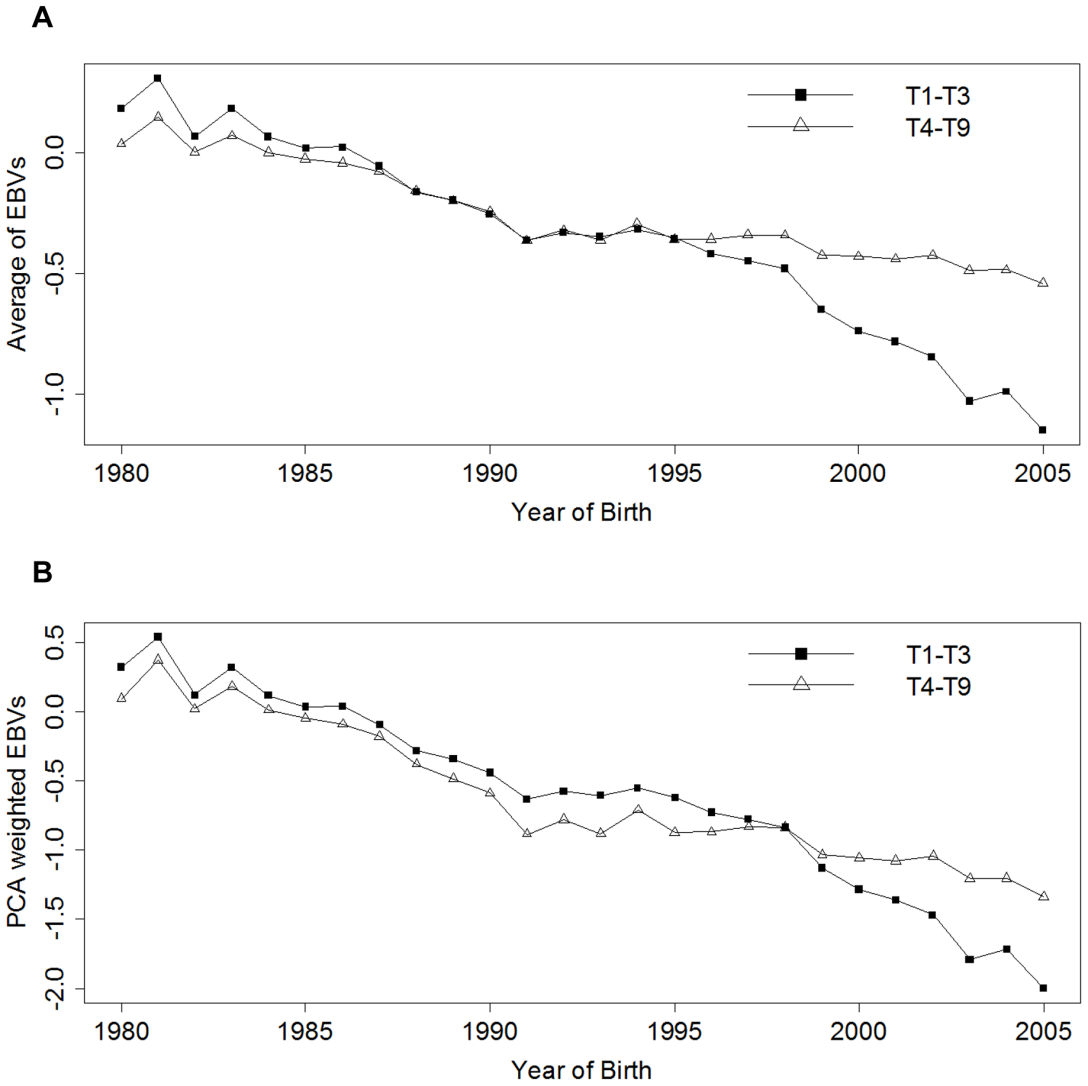
Genetic trend in EBVs of underlying laxity and osteoarthritis traits obtained from combining EBVs by arithmetic means (A) and by first component principal component analysis scores (B) of relevant British Veterinary Association hip traits (BVAHTs) from an Australian cohort of German Shepherd Dogs.

The genetic trend in the BVAHT EBVs has been reported elsewhere and demonstrated significant improvement over time [22]. In the present study, we examined the genetic trend in our combined EBVs to ensure that the laxity and osteoarthritis EBVs created by both estimation methods retained this trend. The resultant trend which is similar to that of the individual BVAHTs reported is shown in Figure 3.

As previously reported in the analysis which showed genetic trend declining (improving) when the EBVs were considered individually [22], considerable genetic improvement is evident in both trait sets over the 22 years portrayed in the graphs. Estimates for 1977–1979 were removed from the Figures due to small numbers of dogs in the cohort for these years. Noting that the graphs indicate genetic trend only (i.e. non-genetic trends -see [5] - are excluded), the steady improvement indicates that selection pressure on BVAHTs was applied effectively by German Shepherd Dog breeders during this time.

### Correlation between laxity and osteoarthritis

**Figure 4:**
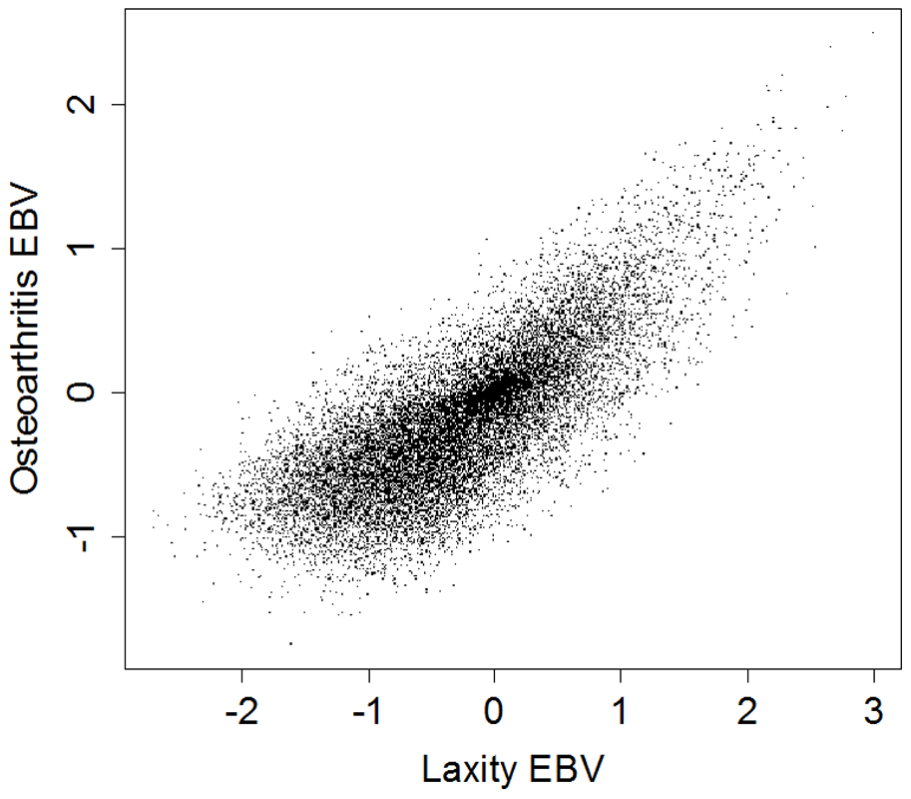
Correlation between the hypothesized Laxity EBV (arithmetic average of EBVs for Norberg angle, Subluxation and Cranial Acetabular Edge) and hypothesized Osteoarthritis EBV (arithmetic mean of remaining British Veterinary Association hip traits) in a cohort of Australian German Shepherd Dogs.

Correlation between laxity EBVs and osteoarthritis EBVs for each dog was 0.771 by the arithmetic average method and 0.775 by the PCA method. The nature of the correlation for the arithmetic method is shown in Figure 4. The extent of the correlation between the EBVs is suggestive that the genetic correlation between the estimates of the underlying traits is considerable.

### Correlation between traits for the litter and individual dog environmental effects

For each of the nine BVAHTs, the estimated variance component for individual dogs exceeded that of the litter variance component; however, between-BVAHT correlations were mostly smaller for the individual dog effects than for the litter effects (see Table 3). The litter-effect correlations show a hint of the same pattern as observed in the genetic correlations between the “laxity” trait group and the “osteoarthritis” trait group. The intra-group average correlations (0.81, 0.90) are higher than the intergroup average correlation (0.75). This pattern is not observed in the individual dog environmental effects.

**Table 3:** Correlation of the individual dog environmental effects (above diagonal) and litter environmental effects (below diagonal) between nine BVA hip traits in a cohort of Australian German Shepherd Dogs.

Correlation	NORB	SUBL	CrAE	DAE	CrEAR	AF	CaAE	FHNE	FHR
**NORB**		0.80	0.74	0.69	0.74	0.66	0.62	0.77	0.75
**SUBL**	0.86		0.69	0.64	0.72	0.64	0.56	0.79	0.72
**CrAE**	0.80	0.77		0.69	0.79	0.64	0.58	0.73	0.70
**DAE**	0.71	0.63	0.76		0.81	0.79	0.77	0.71	0.76
**CrEAR**	0.78	0.74	0.77	0.94		0.77	0.71	0.76	0.77
**AF**	0.55	0.65	0.69	0.82	0.91		0.75	0.75	0.76
**CaAE**	0.73	0.70	0.81	0.99	0.98	0.81		0.69	0.75
**FHNE**	0.77	0.78	0.85	0.72	0.91	0.68	0.84		0.88
**FHR**	0.84	0.86	0.96	1.00	0.99	0.94	0.99	0.98	

NORB =  Norberg Angle; SUBL =  Subluxation; CrAE =  Cranial Acetabular Edge; DAE =  Dorsal Acetabular Edge; CrEAR =  Cranial Effective Acetabular Rim; AF =  Acetabular Fossa; CaAE =  Caudal Acetabular Edge; FHNE =  Femoral Head and Neck Exostosis; FHR =  Femoral Head Remodelling.

## Discussion

Previous work has shown that methodologically sound EBVs for each of the BVAHTs can be calculated for a large cohort of Australian born German Shepherd Dogs. However, in avoiding the combination of the ordinal traits into a summed combined phenotype, the method required the calculation of nine separate EBVs for each animal. Such a large number of EBVs to consider and compare simultaneously would be a severe limitation to utility for dog breeders. The aim of this paper was to explore the underlying genetic correlations between the BVAHTs and suggest a simpler strategy for improving the use of BVAHTs for controlling CHD by the use of EBVs. Ultimately, the authors feel that the trait truly under selection should be hip dysplasia-related welfare. Our ethical responsibility is toward the dog's subjective experience of its own life, despite the formidable difficulties of objectively assessing it, and is only to creating morphologically idealized hips as far as the two accord. Therefore, the genetic correlation of any selection trait or index to a true underlying welfare trait, reflecting all aspects of the animal's subjective experience of its life, should be considered in addition to issues such as the phenotypic variation, heritability, cost of assessment, and extent to which the selection trait is felt to be “causative” of CHD, with each held in appropriate regard. Selection on this trait or index thus chosen can be expected to result in improvements in the welfare trait which is the true goal of selection.

It seems reasonable to suppose that some BVAHTs may be more informative about an animal's future welfare than others. Unfortunately, to the authors' knowledge there is insufficient published information about how predictive each individual BVAHT is of future pain, disease or disability. The deficiency of information in this area is deeply regrettable and should be corrected.

To explore suitable selection indexes, this paper explored the genetic correlations between the BVAHTs in an attempt to elucidate what and how many underlying processes the BVAHTs may be measuring. Earlier work on the correlation of EBVs was suggestive of two processes [22], one measured by traits showing early morphological abnormality or change, which may be a proxy for the extent of hip joint laxity present, and one measured by traits showing later, or more extensive, osteoarthritic change, which may be a proxy for the arthritic response of the joints to this laxity, although the early change traits, especially CrAE, may also, in part, measure pathological response to joint laxity. Our assessment of the genetic correlations, although methodologically imperfect due to use of linear models for ordinal data, added weight to the pattern observed in the correlation of EBVs that various BVAHTs may be measuring one or both of two underlying heritable traits, namely joint laxity and arthritic response to joint laxity. The results of our combined PCA also support this finding.

Lewis et al. [21] found a similar grouping pattern in a large study of Labradors in the United Kingdom, although the genetic correlations found in that population were substantially higher in magnitude. In that study, the average genetic correlation was 0.90 for what we have called Group 1 traits, and 0.85 for what we have called Group 2 traits. Other authors have reported that, when measured by the distraction index, different breeds develop different degrees of arthritis in the presence of a given degree of joint laxity [28], [29]. This *interbreed difference* offers support to the theory that there may be *intrabreed* genetic variation in the development of arthritis in response to laxity. Some additional support for this two-process model may be found in the literature in the form of QTLs which have been found for laxity (as measured by distraction index) [11] and the finding of putative QTLs on four chromosomes for osteoarthritis (as measured by a five-point ordinal macroscopic post-mortem grading) [10], which, as the authors note, were not on the same chromosomes as those which were associated with laxity reported in their 2005 paper [11]. Using a genome-wide association study (GWAS), Zhou et al.[16] reported four SNPs associated with hip dysplasia (as measured by Norberg angle) and two different SNPs associated with hip osteoarthritis (as determined by post mortem analysis or clear osteoarthritic change radiographically) with plausible positional candidate genes.

The authors therefore propose the calculation of an EBV reflecting joint laxity and an EBV reflecting osteoarthritic response to joint laxity for control of hip dysplasia. In this paper, these EBVs were calculated by averaging ordinally derived EBVs for NORB, SUBL and CrAE to calculate an EBV intended to reflect joint laxity, and averaging ordinally derived EBVs for DAE, CrAER, AF, FHNE and FHR to create an EBV intended to reflect osteoarthritic response to dysplastic hip morphology. However there are additional ways in which the EBVs calculated here might be improved to better inform breeders about an animal's genetic value for the underlying process they are intended to reflect. As mentioned above, at the time of the study, the software used was unable to fit multivariate ordinal mixed models. Given the genetic correlation between BVAHTs, multivariate ordinal analysis would have theoretically allowed calculation of ordinal BVAHT EBVs which included information from the other, genetically correlated, BVAHTs, improving the accuracy of the ordinal BVAHT EBVs. Calculating the “laxity” EBV and “osteoarthritis” EBV selection indexes by averaging BVAHT EBVs derived from multivariate analysis in this way could substantially improve the utility of these selection indexes.Further work exploring the utility of multivariate ordinal analysis would be valuable as it would allow more optimal use of the available information. Multivariate linear analysis was considered for the “laxity” EBVs on suitably transformed scores. However, concerns about unwarranted methodological assumptions relating to the linearity of the data and difficulty with obtaining positive definite dispersion matrices when models included more than two BVAHTs meant that this possibility was not further explored in this study.

The utility of the ordinal BHAHT EBVs could also be improved by inclusion of terms representing the veterinarian responsible for the radiograph and the radiologist responsible for reading the radiograph in the model calculating EBVs. These are two potentially significant sources of variation, which were not available in our studies. Additionally, the present authors recommend that in future the actual angle of the Norberg Angle measurement be recorded rather than the ordinal category into which it falls. Given that the angle is measured in any case, reducing the information available to the model by collapsing data into ordinal categories offers no advantage and could represent a significant loss of information and hence decrease in accuracy.

More sensitive measures of laxity, such as dorsolateral subluxation and the distraction index, have been reported in the literature. Zhang et al. [30] reported genetic correlations between a measured Norberg angle and these traits of 0.58 and −0.69 respectively. There may therefore be room for improvement in the estimation of the underlying laxity EBV by including one of these traits if it can be accomplished economically. In livestock industries it is not unusual to use a more easily or cheaply measured trait which is genetically correlated with the trait of interest in a successful program of genetic selection. Additionally, incorporation of genetic marker information into phenotype-based EBVs (sometimes called “marker-assisted selection”) is a relatively standard concept in livestock genetics and has been demonstrated for CHD [13], [15]. While inclusion of traits which are more reflective of the welfare goal of selection would be advantageous, all other things being equal, such a move could be counterproductive if it results in fewer dogs being assessed and therefore less relevant information being available for calculation of EBVs and resulting in a less complete picture of the breed overall. Therefore, new scoring or testing methodologies should be introduced with care and possibly also in parallel with existing methods so that at a minimum, the current level of testing is maintained. With further work it should be possible to incorporate several combinations of results into an EBV-based selection scheme.

In addition to work to improve the extent to which EBVs are truly assessing the animal's genetic value with regard to hip joint “laxity” and propensity to hip “osteoarthritis”, the authors also recommend that further work be undertaken to determine the best way in which to use such EBVs in a selection scheme. As stated, it is more important to maximize the welfare experience of the dog rather than the radiographic appearance of hip joints itself. We therefore recommend that work be undertaken to carefully determine the range of joint laxity and propensity to osteoarthritis that corresponds to our best assessment of whole life welfare of dogs.

Additionally, we recommend exploration of the intra-breed variation of osteoarthritic response to the presence of laxity, and consideration of the utility of identifying alleles (or dogs likely to carry these alleles) which are protective against osteoarthritis for a given amount of joint laxity. The minimum joint laxity which corresponds to acceptable welfare is not currently known. The reason why hip dysplasia is present in multiple breeds and at such high prevalences is not well understood, and unwitting selection pressure applied to increased joint laxity, or a correlated trait, remains a possibility. Additionally, given the moderate genetic correlations between “laxity” and “osteoarthritis” traits and the greater phenotypic variation observed among “laxity” traits, the possibility that the laxity EBV may more accurately predict osteoarthritis-related welfare deficits in late life may also be a possibility. Until the relationship between joint laxity and whole-dog welfare is better understood, a case can be made for preserving alleles which could potentially allow for a greater degree of joint laxity without welfare-deleterious osteoarthritic change.

While such work is undertaken, we recommend that ordinal EBVs for BVAHTs be calculated and averaged into laxity and osteoarthritis EBVs for use, in place of selection based on total BVAHT score. While further research could optimize these EBVs and clarify their appropriate weighting in a selection index, the use of EBVs should improve the accuracy with which an animal's genetic merit is evaluated and the averaging of the EBVs into laxity and osteoarthritis EBVs is our best current understanding of EBVs for the two proposed underlying processes in this population of Australian German Shepherds. PCA has been previously used to combine multiple hip traits into a single index [13]. Although the use of the first component of a principal component analysis gives the best linear combination of scores, the use of the average EBV across traits is recommended for German Shepherd Dogs in Australia, given the extremely high correlation between the two in the data from this population. This recommendation is primarily based upon improving the accessibility and familiarity of the technology to Australian dog breeders who have not used EBV technology before, by not introducing a mathematical process that is likely to be new (and potentially mysterious) to most users, for a very minimal gain. The recommendation is also because the first component weightings may not remain constant over time and changing them over time may create additional confusion and uncertainty toward the technology as EBVs are updated.

While not a single selection index, such as Total BVAHTs scores, provision of two EBVs to breeders, represents a more methodologically defensible combination of the eighteen ordinal BVAHT phenotypes. Breeders already engage with multiple selection criteria with the selection of breeding stock, and the German Shepherd Council of Australia has previously differentiated between scores for different BVAHTs in assessing genetic merit of breeding animals [31], and therefore engaging with CHD as a complex disease process may not be a new experience for many breeders. Provision of two EBVs carries the additional possibility of balancing the two traits within breeding pairs (selection of a mate with complimentary EBVs for a breeding animal with substantially different genetic merit for “laxity” and “osteoarthritis”) and the possibility of guidelines which aim to preserve alleles protective against osteoarthritic change in response to laxity. Even if, in the interim, breeders only engage with the average of the two EBVs the high genetic correlation between the two EBVs should assure improvement in both traits.

The correlations between environmental effects for the BVAHT scores were also estimated in this study. While environmental effects are not directly relevant to a breeding program (beyond accounting for them in the calculation of EBVs), they are very important for canine welfare and investigation into them, such that improved management may lead to reduced disease, is highly desirable. Interestingly, a pattern of higher correlation of non-genetic effects within “laxity” and “osteoarthritis” traits was observed amongst the litter effects, suggesting that different variables among the class of non-genetic litter effects may affect laxity/early morphology and secondary osteoarthritic development differently. This pattern was not observed among the non-genetic individual dog effects, which had smaller and less variable correlations between BVAHTs. Here, “laxity” group correlations were lowest, “osteoarthritis” group correlations were highest and inter-group correlations were intermediate. An interesting difference between the individual and litter environmental effects is the role of the radiographer and radiograph reader. All scores for the individual dog effects are read from the same radiograph, presumably within a short period of time, and may have the potential to affect the radiograph reader's subjective assessments. In contrast, litter mates may not be evaluated within the same session (or the radiograph reader may not notice the relationship between the animals). Therefore correlation in litter environmental effects seems less likely to arise due to radiograph quality concerns or the subjective assessment of the reader.

EBVs offer huge potential in improving the selection and utilization of breeding animals in canine populations [32]. However, it is very important that new selection procedures are evidence-based, flexible enough to include information for genetic markers that may become available and are sufficiently straightforward for dog breeders to utilize rationally. In the regrettable absence of information linking individual BVAHTs to future welfare outcomes, it is recommended utilizing two EBVs for each animal obtained by averaging ordinally-derived EBVs for “laxity” and “osteoarthritic” traits for control of CHD in this population. Additional information in the form of recording examining radiographer, radiographing veterinarian and the actual Norberg angle value have potential to substantially improve the accuracy of EBV selection without much additional expenditure. EBV infrastructure should remain sufficiently flexible to quickly incorporate new information from molecular markers and research into the predictive value of individual BVAHTs and alternate phenotypic assessments of the underlying processes.

## References

[1] Biery DN (2006) The hip joint and pelvis. In: Barr FJ, Kirberger RM, editors. BSAVA Manual of Canine and Feline Musculoskeletal Imaging. Gloucester, UK: British Small Animal Veterinary Association. pp. 119–134.

[2] Morgan JP, Wind A, Davidson AP (2000) Hip Dysplasia. Heriditary Bone and Joint Diseases in the Dog. Hannover, Germany: Shlütersche. pp. 109–208.

[3] Wilson BJ, Nicholas FW, James JW, Wade CM, Tammen I, et al (2011) Symmetry of hip dysplasia traits in the German Shepherd Dog in Australia. Journal of Animal Breeding and Genetics 128: 230–243. 21554417 10.1111/j.1439-0388.2010.00903.x

[4] Smith GK (1997) Advances in diagnosing canine hip dysplasia. Journal of the American Veterinary Medical Association 210: 1451–1457. 9154196

[5] Wilson BJ, Nicholas FW, James JW, Wade CM, Tammen I, et al (2012) Heritability and phenotypic variation of canine hip dysplasia radiographic traits in a cohort of Australian German shepherd dogs. PLoS ONE 7: e39620. 22761846 10.1371/journal.pone.0039620 PMC3384595

[6] Leighton EA (1997) Genetics of canine hip dysplasia. Journal of the American Veterinary Medical Association 210: 1474–1479. 9154200

[7] Janutta V, Hamann H, Distl O (2006) Complex segregation analysis of canine hip dysplasia in German shepherd dogs. Journal of Heredity 97: 13–20. 16267165 10.1093/jhered/esi128

[8] Maki K, Janss LLG, Groen AF, Liinamo AE, Ojala M (2004) An indication of major genes affecting hip and elbow dysplasia in four Finnish dog populations. Heredity 92: 402–408. 14997179 10.1038/sj.hdy.6800434

[9] Friedenberg SG, Zhu L, Zhang Z, Foels W, Schweitzer PA, et al (2011) Evaluation of a fibrillin 2 gene haplotype associated with hip dysplasia and incipient osteoarthritis in dogs. Am J Vet Res 72: 530–540. 21453155 10.2460/ajvr.72.4.530

[10] Mateescu RG, Burton-Wurster NI, Tsai K, Phavaphutanon J, Zhang ZW, et al (2008) Identification of quantitative trait loci for osteoarthritis of hip joints in dogs. American Journal of Veterinary Research 69: 1294–1300. 18828685 10.2460/ajvr.69.10.1294

[11] Todhunter RJ, Mateescu R, Lust G, Burton-Wurster NI, Dykes NL, et al (2005) Quantitative trait loci for hip dysplasia in a crossbreed canine pedigree. Mammalian Genome 16: 720–730. 16245029 10.1007/s00335-005-0004-4

[12] Zhu L, Zhang Z, Feng F, Schweitzer P, Phavaphutanon J, et al (2008) Single nucleotide polymorphisms refine QTL intervals for hip joint laxity in dogs. Animal Genetics 39: 141–146. 18261189 10.1111/j.1365-2052.2007.01691.x

[13] Zhu L, Zhang Z, Friedenberg S, Jung SW, Phavaphutanon J, et al (2009) The long (and winding) road to gene discovery for canine hip dysplasia. The Veterinary Jourmal 181: 97–110. 10.1016/j.tvjl.2009.02.008 PMC2679856 19297220

[14] Chase K, Lawler D, Adler F, Ostrander E, Lark K (2004) Bilaterally asymmetric effects of quantitative trait loci (QTLs): QTLs that affect laxity in the right versus left coxofemoral (hip) joints of the dog (*Canis familiaris*). American Journal of Medical Genetics Part A 124: 239–247. 10.1002/ajmg.a.20363 PMC2778498 14708095

[15] Guo G, Zhou Z, Wang Y, Zhao K, Zhu L, et al (2011) Canine hip dysplasia is predictable by genotyping. Osteoarthritis Cartilage 19: 420–429. 21215318 10.1016/j.joca.2010.12.011 PMC3065507

[16] Zhou Z, Sheng X, Zhang Z, Zhao K, Zhu L, et al (2010) Differential genetic regulation of canine hip dysplasia and osteoarthritis. PLoS ONE 5: e13219. 20949002 10.1371/journal.pone.0013219 PMC2952589

[17] Flückiger M (2007) Scoring radiographs for canine hip dysplasia – the big three organizations in the world. European Journal of Companion Animal Practice 17: 135–140.

[18] Gibbs C (1997) The BVA/KC scoring scheme for control of hip dysplasia: interpretation of criteria. Veterinary Record 141: 275–284. 9316245

[19] Willis MB (1989) *Hip Dysplasia* in *Genetics of the Dog*. London: H.F. & G. Witherby LTD.

[20] Lewis TW, Blott SC, Woolliams JA (2013) Comparative analyses of genetic trends and prospects for selection against hip and elbow dysplasia in 15 UK dog breeds. BMC Genetics 14: 16. 23452300 10.1186/1471-2156-14-16 PMC3599011

[21] Lewis TW, Woolliams JA, Blott SC (2010) Genetic evaluation of the nine component features of hip score in UK labrador retrievers. PLoS ONE 5: e13610. 21042594 10.1371/journal.pone.0013610 PMC2962649

[22] Wilson BJ, Nicholas FW, James JW, Wade CM, Thomson PC (2013) Estimated breeding values for canine hip dysplasia radiographic traits in a cohort of Australian German Shepherd Dogs. PLoS ONE (submitted). 10.1371/journal.pone.0077470 PMC3812223 24204838

[23] Falconer DS, Mackay TFC (1996) Introduction to Quantitative Genetics- 4th Edition. Harlow, England: Pearson Education Limited.

[24] Gilmour AR, Gogel BJ, Cullis BR, Thompson R (2006) ASReml User Guide Release 2.0. Hemel Hempstead, HP1 1ES, UK: VSN International Ltd.

[25] Patterson HD, Thompson R (1971) Recovery of interblock information when block sizes are unequal. Biometrika 58: 545–554.

[26] Gunawan B, James JW (1986) The Use of ‘Bending’ in Multiple Trait Selection of Border Leicester-Merino Synthetic Populations. Australian Journal of Agricultural Research 37: 539–547.

[27] Hayes JF, Hill WG (1981) Modification of Estimates of Parameters in the Construction of Genetic Selection Indices(‘Bending’). Biometrics 37: 483–493.

[28] Popovitch CA, Smith GK, Gregor TP, Shofer FS (1995) Comparison of susceptibility for hip dysplasia between Rottweilers and German shepherd dogs. Journal of the American Veterinary Medical Association 206: 648–650. 7744685

[29] Smith GK, Mayhew PD, Kapatkin AS, McKelvie PJ, Shofer FS, et al (2001) Evaluation of risk factors for degenerative joint disease associated with hip dysplasia in German Shepherd Dogs, Golden Retrievers, Labrador Retrievers, and Rottweilers. Journal of the American Veterinary Medical Association 219: 1719–1724. 11767921 10.2460/javma.2001.219.1719

[30] Zhang ZW, Zhu L, Sandler J, Friedenberg SS, Egelhoff J, et al (2009) Estimation of heritabilities, genetic correlations, and breeding values of four traits that collectively define hip dysplasia in dogs. American Journal of Veterinary Research 70: 483–492. 19335104 10.2460/ajvr.70.4.483

[31] Hedberg K (2000) Review of the HD Scheme – NBC Meeting August 2000. http://www.gsdcouncilaustralia.org/review_of_hd_scheme_2000.doc (Accessed 15/3/2013).

[32] Thomson PC, Wilson BJ, Wade CM, Shariflou MR, James JW, et al (2010) The utility of estimated breeding values for inherited disorders of dogs. Veterinary Journal 183: 243–244. 10.1016/j.tvjl.2009.12.002 20042354

